# A prospective randomised trial to study the role of levamisole and interferon alfa in an adjuvant therapy with 5-FU for stage III colon cancer

**DOI:** 10.1038/sj.bjc.6602555

**Published:** 2005-04-26

**Authors:** W Schippinger, M Jagoditsch, C Sorré, M Gnant, G Steger, H Hausmaninger, B Mlineritsch, R Schaberl-Moser, H J Mischinger, F Hofbauer, P Holzberger, M Mittlböck, R Jakesz

**Affiliations:** 1 Medical Department, Division of Oncology, Graz Medical School, A-8036 Graz, Austria; 2 St Veit Hospital, A-9300 St Veit/Glan, Austria; 3 Department of Surgery, Vienna Medical School, A-1090 Vienna, Austria; 4 First Medical Department, Division of Oncology, Vienna Medical School, A-1090 Vienna, Austria; 5 Third Medical Department of the Private Medical Paracelsus University Salzburg, A-5020 Salzburg, Austria; 6 Department of Surgery, Graz Medical School, A-8036 Graz, Austria; 7 Department of Surgery, Oberpullendorf Hospital, A-7350 Oberpullendorf, Austria; 8 Department of Surgery, Innsbruck Medical School, A-6020 Innsbruck, Austria; 9 Core Unit for Medical Statistics and Informatics, Vienna Medical School, A-1090 Vienna, Austria

**Keywords:** colon cancer, adjuvant therapy, levamisole, interferon

## Abstract

The purpose of this trial was to examine the efficacy of the addition of levamisole (LEV) or interferon alfa (IFN) to an adjuvant chemotherapy with 5-fluorouracil (5-FU) in patients with stage III colon cancer. According to a 2 × 2 factorial study design, 598 patients were randomly assigned to one of four adjuvant treatment arms. Patients in arm one received 5-FU weekly for 1 year, patients in arm two 5-FU plus LEV, in arm three 5-FU plus IFN and patients in arm four 5-FU, LEV and IFN. The relative risk of relapse and the relative risk of death were significantly higher for patients treated with LEV compared with those without LEV treatment (HR 1.452, 95% CI 1.135–1.856, *P*=0.0028; HR 1.506, 95% CI 1.150–1.973, *P*=0.0027, respectively). No significant impact on survival was observed for therapy with IFN in the univariate analysis. The addition of LEV to adjuvant 5-FU significantly worsened the prognosis of patients with stage III colon cancer. Interferon alfa had no significant influence on survival when combined with adjuvant 5-FU, but increased the toxicity of therapy substantially.

Colorectal cancer is the second most common malignant disease in developed countries (Parkin *et al*, 2001). About 50% of patients with colorectal cancer who undergo surgical treatment subsequently develop recurrent disease ultimately leading to incurability. Adjuvant chemotherapy containing 5-fluorouracil (5-FU) after complete surgical removal of the primary tumour has been shown in randomised trials to decrease the risk of disease relapse (Wolmark *et al*, 1988; Laurie *et al*, 1989). Based on reports suggesting a possible benefit of combining 5-FU with levamisole (LEV), an anthelmintic drug with immunomodulatory activity, a large Intergroup trial was launched in 1984 comparing 5-FU plus LEV for 1 year with observation in patients with curatively resected stage III colon cancer. 5-Fluorouracil with LEV reduced the risk of cancer recurrence by 41% and the overall death rate by 33% (Moertel *et al*, 1990). In conclusion of these results, 5-FU and LEV were considered to be standard adjuvant treatment for stage III colon cancer for several years (NIH, 1990). However, other authors failed to show a benefit by adding LEV to adjuvant 5-FU chemotherapy (Sertoli *et al*, 1987), and LEV as a single-agent therapy has been shown to be ineffective as adjuvant therapy (Arnaud *et al*, 1989). In addition, the combination of LEV with 5-FU in advanced colon cancer was shown not to be superior to 5-FU single-agent therapy (Buroker *et al*, 1985). At the time the present study was initiated, combination of LEV and 5-FU was considered to be standard therapy to eliminate minimal residual disease. Consequently, this trial was designed to evaluate this combination treatment in comparison with an experimental approach in patients with stage III colon cancer.

One way to enhance the antitumour activity of 5-FU was the implementation of interferon alfa (IFN) in treatment strategies of colorectal cancer. Interferon alfa as an immunomodulatory cytokine had been shown to increase the cytotoxic activity of 5-FU in human cancer cell lines (Wadler *et al*, 1990). Several clinical trials confirmed an increased responsiveness by addition of IFN to 5-FU in the treatment of metastatic colon cancer (Wadler *et al*, 1989, 1991). According to these promising data, the second aim of the present study was to examine the efficacy of IFN plus 5-FU as adjuvant therapy for stage III colon cancer in a 2 × 2 factorial study design.

## MATERIALS AND METHODS

This multicentre trial was initiated by the Austrian Breast and Colorectal Cancer Study Group (ABCSG) and involved 41 participating hospitals. The protocol was approved by the ethics committees of the participating institutions. Enrolment of patients began in October 1991 and was completed in April 1999.

### Patient selection

To be eligible, patients had to fulfil the following inclusion criteria: histologically proven carcinoma of the colon with lymph node metastasis (stage III tumour according to the International Union Against Cancer); potentially curative resection without gross or microscopic evidence of residual disease; age between 18 and 80 years; WHO performance status of 0 or 1; absence of severe concomitant disease and other malignancies; and adequate bone marrow function. The following were considered as exclusion criteria: prior or concomitant chemotherapy, immunotherapy and radiotherapy, carcinoma of the rectum defined as tumour below the anatomical rectosigmoidal border line or within 16 cm from the anal verge measured by a nonflexible rectoscope. Other exclusion criteria were the presence of metastasis or a time period of more than 42 days from surgery to start of adjuvant therapy.

Written informed consent was obtained from all patients participating in this trial.

Site visits were performed in regular intervals to check the original data concerning eligibility and to review the documented chemotherapy and follow-up data.

### Surgical treatment

A tumour of the caecum, the ascending colon or the hepatic flexure was resected with a right hemicolectomy with resection of the right colic artery and the ileocolic artery. A tumour of the colon transversum was treated by resection of the appropriate part of the colon including the hepatic and splenic flexure. Carcinomas of the descending colon were resected by a left hemicolectomy including the left colic artery. Finally, sigmoid tumours were treated by a sigmoid resection including the inferior mesenteric blood vessels. The resection of at least seven regional lymph nodes was recommended.

### Randomisation and stratification procedures

Patients were stratified by sex, age (⩽65 *vs* >65–80 years), tumour size (T1 *vs* T2 *vs* T3 *vs* T4), number of involved lymph nodes (⩽3 *vs* >3 lymph nodes) and tumour differentiation (G1/2 *vs* G3/4). Randomisation was performed by computer, assigning the patients according to a 2 × 2 factorial study design to one of four postoperative treatment arms: (1) 5-FU, (2) 5-FU plus LEV, (3) 5-FU plus IFN and (4) 5-FU plus LEV plus IFN according to the method of Pocock and Simon (1975), which marginally balances the following criteria: sex, age (⩽65 *vs* >65–80 years), tumour size (T1 *vs* T2 *vs* T3 *vs* T4), number of involved lymph nodes (⩽3 *vs* >3 lymph nodes), tumour differentiation (G1/2 *vs* G3/4) and state.

### Schedules of chemotherapy administration

Adjuvant chemotherapy was required by the trial protocol to be started within 42 days after tumour resection. In treatment arm 1, adjuvant therapy consisted of 5-FU 450 mg m^−2^ as an intravenous bolus given initially on five consecutive days and then once weekly, starting on day 28 for 48 weeks (=52 weeks of therapy). Patients in treatment arm 2 received 5-FU as in arm 1 and additionally LEV 50 mg orally three times a day for three consecutive days (days 1–3) every 14 days. In arm 3, patients received 5-FU as in arm 1 and interferon alfa-2C (Berofor®, Boehringer Ingelheim GmbH, Germany) at a dose of 3.5 × 10^6^ U subcutaneously three times a week (Monday, Wednesday, Friday) for 52 weeks. Arm 4 consisted of 5-FU plus LEV in the same schedule as in arm 2 and of IFN 3.5 × 10^6^ U subcutaneously three times a week (Monday, Wednesday, Friday) for 52 weeks.

In all four treatment arms, dose modifications were required for haematologic or other severe toxicity. According to the protocol, the 5-FU dosage was reduced by 25% when patients developed leucopenia (3.0–3.5 × 10^9^ l^−1^) and/or thrombocytopenia (<100 × 10^9^ and >75.10^9^ l^−1^). The dose was reduced by 50%, when thrombocyte counts decreased to 50 × 10^9^–75 × 10^9^ l^−1^. In case of more severe leucopenia (<3.0 × 10^9^ l^−1^) and/or thrombocytopenia (<50 × 10^9^ l^−1^), treatment was delayed for 1 week.

### Follow-up

During the first year after randomisation (=treatment period), patients were evaluated every 3 months, during years 2–5 every 6 months and thereafter, once yearly until year 10 after randomisation. A physical examination with evaluation of body weight and performance status, determination of blood count, serum liver function parameters, carcinoembryonic antigen (CEA) and tests for occult blood in stool, as well as chest radiography and ultrasonography of the liver were performed at each follow-up visit. Colonoscopy was required during the first 5 years every 6 months, then once a year.

### Statistical methods

The trial was planned as 2 × 2 factorial design with four arms (LEV *vs* no LEV and IFN *vs* no IFN). It was designed to detect a difference in 5-year survival rate for each factor of 12% (59–71%) with a power of 85% and a two-sided significance level of 0.05, so that approximately 200 patients would be recruited for 3 years and followed-up for another 4 years or equivalently 225 observed deaths.

The intention-to-treat analysis included all eligible patients for whom complete baseline data were available. All patient data were collected at the study group's central data office and processed and analysed applying SAS software (SAS Institute, Cary/NC, USA). Distribution of prognostic factors to the treatment arms was described with frequencies for categorical variables and with medians for continuous data and tested with the *χ*^2^-test and Kruskal–Wallis test, respectively.

Overall survival (OS) was defined as the time between date of randomisation of primary colon cancer until date of last visit or date of death, independent of the cause of death. Disease-free survival (DFS) was defined as time from randomisation to either recurrence of colon cancer, occurrence of metastases, occurrence of a second primary cancer, or death without evidence of recurrence or a second primary tumour, or date of last visit.

Overall survival and DFS were estimated and graphically presented according to the method of Kaplan and Meier (1958). Differences between curves were assessed by the Mantel log-rank test for censored survival data (Mantel, 1966). The Cox proportional hazards model (Cox, 1972) was used to assess the prognostic values of treatment, sex, age, lymph node status, tumour grading, tumour stage, tumour localisation and number of surgically removed lymph nodes in univariate and multiple analyses and quantifies their effect by hazard ratios. Corresponding 95% confidence intervals are also given. Furthermore, it was applied to assess interactions between treatment and the other covariates and to detect nonproportionality. A Cox regression model with the time-dependent factor was modelled to assess the effect of the cumulative 5-FU dose, which increases with time from the first to the last treatment delivery. All *P*-values given are two-sided.

## RESULTS

### Patient characteristics

A total of 598 patients entered this prospective randomised trial. A total of 22 erroneously randomised patients (3.7%) were found to be ineligible and were therefore not included in the analysis. Four patients were removed from the statistical analysis for lack of any baseline or follow-up data. Statistical analyses were performed with the data of the remaining 572 eligible patients according to the intention-to-treat principle. Baseline patient and tumour characteristics are reported in Table 1. The patient and tumour characteristics were well balanced between the four treatment arms. The median follow-up time for the study population was 84.3 months and 50% of the patients had a follow-up time of 65.2–108.2 months.

### Treatment adherence and delivered 5-FU dose

Adjuvant therapy as planned in the protocol was prematurely discontinued without evidence of tumour relapse in 25 patients (17.2%) of the 5-FU single-agent group, in 29 patients (20.7%) of the 5-FU plus LEV group, in 52 patients (36.6%) of the 5-FU plus IFN group and in 72 patients (49.7%) of the 5-FU/LEV/IFN triple-agent group. In most of the cases, the reasons for treatment discontinuation were toxicity and the patients' wish to discontinue therapy. The median, actually delivered 5-FU dose (5-FU dose given in grams per square metre) cumulated over time was 22.397 g m^−2^ in treatment arm 1, 21.600 g m^−2^ in arm 2, 17.118 g m^−2^ in therapy arm 3 and 13.500 g m^−2^ in arm 4.

The median cumulatively delivered 5-FU dose was significantly lower in the therapy arms including LEV than in the treatment arms without LEV (17.953 *vs* 20.828 g m^−2^, *P*=0.0055) and also significantly lower in the therapy arms including IFN compared to the treatment arms without IFN (15.647^2^ *vs* 22.050 g m^−2^, *P*<0.0001).

As older patients may tend to have lower tolerability for treatment combinations, the association between age and cumulatively delivered 5-FU dose (<80 *vs* ⩾80% of planned dose) was investigated in a cross-table. The results showed a trend to higher 5-FU dose for younger patients in treatment arms 1 and 2, where no IFN was applied. The two arms with IFN treatment (groups 3 and 4) showed a higher rate of patients with lower cumulatively delivered 5-FU dose; however, lower 5-FU dose seemed not to be related to age of patients.

### Tumour relapse and disease-free survival

To date, 259 patients have relapsed. Tumour relapse was documented in 136 of 285 patients (47.7%) in the therapy arms containing LEV (treatment arms 2 and 4), in 113 of 287 patients (39.4%) in the therapy arms not containing LEV (arms 1 and 3), in 134 of 287 patients (46.7%) treated with IFN (arms 3 and 4) and in 125 of 285 patients (43.8%) not treated with IFN (arms 1 and 2).

In univariate analyses, the relative risk of relapse in the patient cohort treated with LEV (treatment arms 2 and 4) was significantly higher than in the patient group that had not received LEV (arms 1 and 3) (HR 1.452, 95% CI 1.135–1.856, log-rank test: *P*=0.0028). There was no statistically significant difference in the relative risk of relapse between the patient group treated with IFN and the group without IFN treatment (HR 1.019, 95% CI 0.798–1.300, log-rank test: *P*=0.8826).

Consequently, DFS was significantly shorter for patients treated with LEV compared to patients who had not received LEV.

No significant difference in DFS was observed between patients treated with IFN and patients treated without IFN.

**Figure 1:**
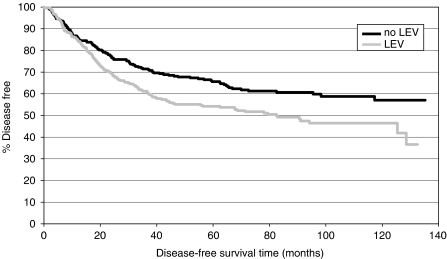
Disease-free survival: LEV *vs* no LEV.

Kaplan–Meier curves for DFS are shown in Figures 1 and 3.

### Overall survival

Altogether, 217 of 572 eligible patients have died: 126 of 285 patients (44.2%) in the therapy arms containing LEV (treatment arms 2 and 4), 91 of 287 patients (31.7%) in the therapy arms not containing LEV (arms 1 and 3), 114 of 287 patients (39.7%) treated with IFN (arms 3 and 4) and 103 of 285 patients (36.1%) not treated with IFN (arms 1 and 2).

Univariate analyses revealed that patients treated with LEV had a significantly higher relative risk of death compared to those patients who were not treated with LEV (HR 1.506, 95% CI 1.150–1.973, *P*=0.0027).

No significant difference in the relative risk of death was observed between the patient group treated with IFN and the group treated without IFN (HR 1.046, 95% CI 0.802–1.366, *P*=0.738).

Considering these data, it follows that patients treated with LEV had a significant disadvantage in OS compared to those patients who did not receive LEV.

There was no significant difference in OS between patients treated with IFN and the patients without IFN therapy.

**Figure 2:**
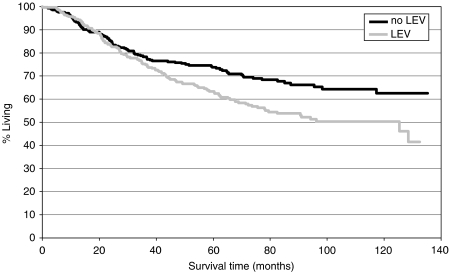
Overall survival: LEV *vs* no LEV.

Kaplan–Meier curves for OS are presented in Figures 2 and 4.

### Multiple analyses of prognostic factors

Cox regression analysis revealed age, sex, tumour size, the number of metastatic lymph nodes and treatment with LEV as significant prognostic factors for DFS. Therapy with IFN, grading and tumour localisation had no significant impact on DFS in the multiple analysis.

With regard to OS, sex, tumour size, the number of metastatic lymph nodes and again treatment with LEV were found to be significant prognostic variables. Therapy with IFN, grading, tumour localisation and age demonstrated no significant impact on OS in the Cox model. The results of the multiple analyses are shown in Tables 2 and 3.

An additional Cox analysis including the cumulatively delivered 5-FU dose per patient demonstrated this parameter to have a statistically significant impact on OS (*P*<0.0001), but no significant influence on DFS (*P*=0.1232). Adjusting for the cumulative 5-FU dose in the multiple analyses for DFS, the effect of IFN remained nonsignificant and the effect of LEV also remained approximately the same. Analogous multiple analyses for OS revealed that the effect of IFN was significant (HR 0.730, *P*=0.0347). The effect of LEV was slightly weaker (HR 1.334, *P*=0.0418) when adjusted for the cumulative 5-FU dose but remained statistically significant (all patients were considered in these analyses according to the intention-to-treat principle). When considering only patients who received at least one therapy cycle, three patients were excluded from the analyses and the effect of LEV on OS again remained significant.

**Figure 3:**
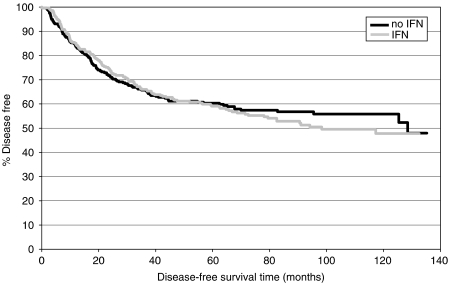
Disease-free survival: IFN *vs* no IFN.

**Figure 4:**
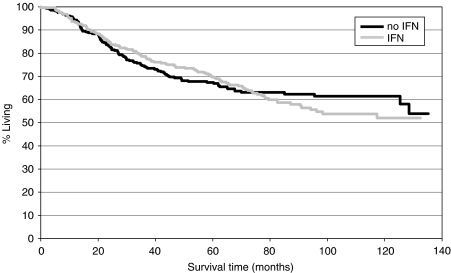
Overall survival: IFN *vs* no IFN.

Analysing interaction terms with respect to time, the multiple model revealed treatment with IFN as a time-dependent prognostic factor for DFS and OS, indicating that therapy with IFN reduces the risk of relapse and the risk of death temporarily, but not over the entire follow-up period (see Figures 3 and 4).

### Toxicity

Grade 3 and 4 toxicities were documented during adjuvant therapy in 11.2% of the patients in the 5-FU single-agent arm, in 21.5% of the patients treated with 5-FU/LEV, in 26.7% of the patients in the 5-FU/IFN arm and in 40.4% of the patients treated in the 5-FU/LEV/IFN arm.

The distribution of grade 3 and 4 toxic effects is shown in Table 4.

## DISCUSSION

At the time of initiation of this trial, the combination of 5-FU with LEV was considered as the standard adjuvant treatment of stage III colon cancer. However, in the final phase of recruitment to this trial, data from other studies showed that neither LEV nor IFN is of benefit in the adjuvant treatment of colon cancer. Final analysis data of the Intergroup trial INT 0089 suggested 5-FU plus leucovorin for 6 months to be preferable over 12 months of 5-FU plus LEV for reasons of toxicity, costs and patients convenience (Haller *et al*, 1998). The NSABP trial C-04 compared the efficacy of 5-FU plus leucovorin with 5-FU plus LEV and with the triple combination of 5-FU plus leucovorin plus LEV. The study demonstrated a statistically significant prolongation of DFS in favour of 5-FU plus leucovorin compared to 5-FU plus LEV, but no significant difference between these two treatment arms in terms of OS. The comparison between patients treated with 5-FU plus leucovorin and those who received 5-FU plus leucovorin plus LEV showed no difference for either DFS or OS (Wolmark *et al*, 1999). The NSABP protocol C-05 evaluated the efficacy of the addition of interferon alfa-2a to 5-FU and leucovorin in the adjuvant treatment of colon cancer. The results of this trial showed no significant difference either in DFS or in OS for the IFN arm (Wolmark *et al*, 1998). Owing to the absence of significant differences in OS in these trials examining the role of LEV and IFN in the adjuvant setting of colon cancer, continuation of accrual in the present trial was considered as ethically justifiable until the end of the accrual period in 1999.

In this trial, it was documented for the first time that the combination of LEV with 5-FU as adjuvant treatment of stage III colon cancer is significantly inferior in terms of DFS and OS compared to a therapy with 5-FU alone. Owing to the finding that patients in the LEV-containing therapy arms received less cumulative 5-FU, the unfavourable effect of LEV could be assumed to be caused by the lower 5-FU dose only. However, the multiple analysis adjusting for differences in the cumulatively delivered 5-FU dose in the treatment groups caused only a minor change of observed hazard ratio for the therapy effect of LEV. Thus, it can be ruled out that the observed differences in DFS and OS are exclusively a consequence of different 5-FU doses in the treatment arms. Levamisole affects survival independently of the delivered cumulative 5-FU dose. These findings contribute to assess the efficacy of LEV in 5-FU-based adjuvant therapy, since the Intergroup trial published by Moertel *et al* (1990), which led to the NIH consensus suggesting 5-FU and LEV as standard treatment for stage III colon cancer, compared 5-FU plus LEV with observation and with LEV alone. The Intergroup trial revealed, for the single-agent LEV arm, an identical OS to no adjuvant therapy. Another study addressing the role of LEV in colon cancer, the Quick and Simple and Reliable (QUASAR) trial, investigated a possible survival benefit by using a higher dose of folinic acid or by addition of LEV to 5-FU and folinic acid and found no survival benefit from LEV (QUASAR Collaborative Group, 2000). Patients treated with LEV had an even higher, although statistically not significant, odds ratio for disease recurrence and death compared to patients without LEV therapy. The observed trend to deterioration of prognosis by addition of LEV in the QUASAR trial is not concordant with the findings of our study, which shows a significantly detrimental effect of LEV in 5-FU-based adjuvant therapy of colon cancer. A possible explanation for the more distinct survival disadvantage for the LEV arm in the present study could be the higher 5-FU dose density in a fraction of the QUASAR study population, whereas the dose density of LEV was the same as in the present study.

In view of the findings of the present study, an antagonising effect of LEV on 5-FU can be suggested. Furthermore, the findings of this trial correlate very well with the results of a recently published preclinical study, which found that LEV did not only lack a significant growth inhibitory effect as a single agent in colon cancer cell lines, but also antagonised 5-FU-induced tumour growth inhibition when combined with this cytostatic agent (Wiebke *et al*, 2003).

The results of the present study imply that the addition of LEV to 5-FU in the adjuvant treatment of colon cancer has an unfavourable effect on patient prognosis and should therefore not be considered as a possible adjuvant treatment option.

A smaller trial comparing an adjuvant therapy with 5-FU plus LEV for 1 year with 5-FU plus folinic acid, administered for either 6 or 12 months, showed that 6 or 12 months of treatment with 5-FU plus folinic acid is equivalent to 12 months with 5-FU plus LEV (Dencausse *et al*, 2002).

Another study comparing 5-FU plus leucovorin with 5-FU plus LEV, both for 1 year as adjuvant therapy in patients with resected stage III colon cancer, showed a significant superiority of the 5-FU plus leucovorin arm in terms of DFS and OS (Arkenau *et al*, 2003).

The INTACC trial investigating whether the addition of leucovorin to the combination of 5-FU plus LEV improves survival in patients with Dukes' B and C colon cancer showed no statistical difference in DFS and OS between the 5-FU/LEV and the 5-FU/LEV/leucovorin treatment arms (Di Costanzo *et al*, 2003).

The second goal of the present study was to examine the effect of IFN in a 5-FU-based adjuvant therapy for stage III colon cancer. Although Kaplan–Meier curves suggest a survival advantage within the first years of follow-up for patients treated with IFN, this early benefit in terms of DFS and OS is counterbalanced by a higher number of disease relapses and deaths at later periods of follow-up. Higher toxicity and a higher frequency of premature therapy cessations resulted in a significantly lower cumulative 5-FU delivery in the IFN treatment arms, which could be a reason for the missing effect of IFN on DFS and OS. In fact, the multiple model adjusting for the delivered 5-FU dose demonstrates a significantly positive impact of IFN on OS, but not on DFS. As 5-FU dose is different between therapy groups, it cannot be ruled out that patients receiving a higher 5-FU dose are a selected group with good prognostic factors. Thus, the improved OS hazard ratio for the IFN treatment group in the 5-FU adjusted model could be explained by age or other unobserved nontreatment-related prognostic factors also. These findings confirm the results of other trials that the addition of IFN to 5-FU does not confer a survival benefit (Wolmark *et al*, 1998).

Frequency of grade 3 and 4 toxicities was increased in therapy arms including LEV or IFN. The highest rate of severe toxicities was documented within the patient group treated with the triple-agent combination. Premature discontinuation of therapy was most frequent in the treatment arms containing IFN; the highest number of therapy cessations was observed in the 5-FU/LEV/IFN arm.

Summarising the results of this study, neither LEV nor IFN showed efficacy in a 5-FU-based adjuvant therapy for stage III colon cancer.

## Figures and Tables

**Table 1:** Clinical and pathological characteristics by treatment arms

	**5-FU (%)**	**5-FU+LEV (%)**	**5-FU+IFN (%)**	**5-FU+LEV+IFN (%)**	***P*-value**
Number of patients	145	140	142	145	
Median age (years)	63.0	62.6	62.9	63.2	0.7249
Male/female	51.7/48.3	50.7/49.3	51.4/48.6	50.3/49.7	0.9953

*T category*					0.9897
T1	2.1	0.7	1.4	0.7	
T2	8.3	7.9	9.2	9.7	
T3	75.2	75.0	73.2	73.8	
T4	14.5	16.4	16.2	15.8	

*N category*					0.9991
N1	55.8	55.0	54.9	56.5	
N2	29.0	29.3	31.0	28.3	
N3	15.2	15.7	14.1	15.2	

*Grading*					0.9616
G1 and G2	67.6	69.1	70.4	69.7	
G3 and G4	32.4	30.9	29.6	30.3	

*Location of tumour*					0.4467
Caecum and right colon	30.3	32.9	25.4	29.0	
Left colon and sigmoid	46.9	46.4	48.6	54.5	
Flexures and transverse colon	22.8	20.7	26.1	16.5	

5-FU=5-fluorouracil; LEV=levamisole; IFN=interferon alfa.

**Table 2:** Multiple analysis: evaluation of prognostic factors for DFS

	**HR**	**95% CI**	***P*-value**
Age	1.134	1.011–1.273	0.0325
Sex	1.331	1.036–1.711	0.0255
Tumour size	1.432	1.119–1.831	0.0043
Number of metastatic lymph nodes	1.369	1.058–1.771	0.0169
Histopathological grading	1.241	0.950–1.621	0.1132

*Location of tumour*			0.8169
Left colon and sigmoid	1.095	0.814–1.472	
Flexures and transverse colon	1.097	0.766–1.571	
Treatment with LEV	1.430	1.115–1.835	0.0049
Treatment with IFN	0.942	0.736–1.206	0.6364

For the HR, the reference category for categorical covariates was female for sex, ⩽3 metastatic lymph nodes for number of metastatic lymph nodes, G1/G2 for histopathological grading, caecum and right colon for location of tumour, no LEV for treatment with LEV and no IFN for treatment with IFN. DFS=disease-free survival; LEV=levamisole; IFN=interferon alfa.

**Table 3:** Multiple analysis: evaluation of prognostic factors for OS

	**HR**	**95% CI**	***P*-value**
Age	1.011	0.998–1.025	0.0923
Sex	1.452	1.103–1.912	0.0078
Tumour size	1.659	1.266–2.174	0.0002
Number of metastatic lymph nodes	1.417	1.069–1.879	0.0155
Histopathological grading	1.232	0.920–1.650	0.1623

*Location of tumour*			0.7833
Left colon and sigmoid	1.121	0.812–1.548	
Flexures and transverse colon	1.065	0.715–1.585	
Treatment with LEV	1.460	1.110–1.920	0.0069
Treatment with IFN	0.975	0.744–1.278	0.8539

For the HR, the reference category for categorical covariates was female for sex, ⩽3 metastatic lymph nodes for number of metastatic lymph nodes, G1/G2 for histopathological grading, caecum and right colon for location of tumour, no LEV for treatment with LEV and no IFN for treatment with IFN. OS=overall survival; LEV=levamisole; IFN=interferon alfa.

**Table 4:** Patients with grade 3 and 4 toxicities in the four treatment arms

	**5-FU (%)**	**5-FU+LEV (%)**	**5-FU+IFN (%)**	**5-FU+LEV+IFN (%)**	***P*-value**
Nausea	3.5	6.5	3.6	8.2	0.2197
Diarrhoea	3.5	3.6	2.9	3.4	0.9885
Stomatitis	0	2.2	0.7	1.4	0.2870
Fever	0	0.7	2.2	4.1	0.0331
Leucopenia	0.7	1.4	3.6	11.6	<0.0001
Thrombopenia	0.7	1.4	2.2	1.4	0.8806
Alopecia	0	0.7	0.7	1.4	0.7606
Other toxic effects	2.8	5.0	10.8	8.9	0.0488

5-FU=5-fluorouracil; LEV=levamisole; IFN=interferon alfa.
